# ICU strain and outcome in COVID-19 patients—A multicenter retrospective observational study

**DOI:** 10.1371/journal.pone.0271358

**Published:** 2022-07-19

**Authors:** Alexandre Demoule, Muriel Fartoukh, Guillaume Louis, Elie Azoulay, Safaa Nemlaghi, Edouard Jullien, Cyrielle Desnos, Sebastien Clerc, Elise Yvin, Nouchan Mellati, Cyril Charron, Guillaume Voiriot, Yoann Picard, Antoine Vieillard-Baron, Michael Darmon

**Affiliations:** 1 Service de Médecine Intensive et Réanimation (Département R3S), AP-HP, Groupe Hospitalier Universitaire APHP-Sorbonne Université, site Pitié-Salpêtrière, Paris, France; 2 INSERM, UMRS1158 Neurophysiologie Respiratoire Expérimentale et Clinique, Sorbonne Université, Paris, France; 3 Service de Médecine Intensive et Réanimation, AP-HP, Groupe Hospitalier Universitaire APHP-Sorbonne Université, site Tenon, Paris, France; 4 Groupe de Recherche Clinique CARMAS, Université Paris Est Créteil, Créteil, France; 5 Service de Réanimation Polyvalente, Hôpital de Mercy, CHR Metz-Thionville, Metz, France; 6 APHP, Hôpital Saint-Louis, Medical Intensive Care Unit, ECSTRA team, and Clinical Epidemiology, UMR 1153, Center of Epidemiology and Biostatistics, CRESS, INSERM, Université de Paris, Paris, France; 7 Service de Médecine Intensive et Réanimation, AP-HP, Groupe Hospitalier Universitaire PARIS-SACLAY, Site Ambroise Paré, DMU CORREVE, Boulogne Billancourt, France; 8 INSERM UMR 1018, Clinical Epidemiology Team, CESP, Université de Paris Saclay, Boulogne Billancourt, France; University of Palermo, ITALY

## Abstract

**Purpose:**

To compare the characteristics, management, and prognosis of patients admitted to intensive care units (ICU) for coronavirus disease (COVID)-19 during the first two waves of the outbreak and to evaluate the relationship between ICU strain (ICU demand due to COVID-19 admissions) and mortality.

**Methods:**

In a multicentre retrospective study, 1166 COVID-19 patients admitted to five ICUs in France between 20 February and 31 December 2020 were included. Data were collected at each ICU from medical records. A Cox proportional-hazards model identified factors associated with 28-day mortality.

**Results:**

640 patients (55%) were admitted during the first wave (February to June 2020) and 526 (45%) during the second wave (July to December 2020). ICU strain was lower during the second wave (-0.81 [-1.04 –-0.31] vs. 1.18 [-0.34–1.29] SD when compared to mean COVID-19 admission in each center during study period, P<0.001). Patients admitted during the second wave were older, had more profound hypoxemia and lower SOFA. High flow nasal cannula was more frequently used during the second wave (68% vs. 39%, P<0.001) and intubation was less frequent (46% vs. 69%, P<0.001). Neither 28-day mortality (30% vs. 26%, P = 0.12) nor hospital mortality (37% vs. 31%, P = 0.27) differed between first and second wave. Overweight and obesity were associated with lower 28-day mortality while older age, underlying chronic kidney disease, severity at ICU admission as assessed by SOFA score and ICU strain were associated with higher 28-day mortality. ICU strain was not associated with hospital mortality.

**Conclusion:**

The characteristics and the management of patients varied between the first and the second wave of the pandemic. Rather than the wave, ICU strain was independently associated with 28-day mortality, but not with hospital mortality.

## Introduction

About 4% of severe acute respiratory syndrome coronavirus 2 (SARS-CoV-2) infections require intensive care unit (ICU) admission [1–6], mostly for severe pneumonia causing acute respiratory failure [3, 6, 7]. The first wave of the outbreak was declared in France in February 2020. Intensivists faced an unprecedented number of patients requiring ventilatory support, and were faced with two major challenges. The first one was the discovery of a previously unknown disease and its specific management. The second one was a dramatic surge of patients with the risk of ICUs becoming overwhelmed. Many large cohort studies have extensively described the characteristics, management and outcome of patients admitted during this first wave of the pandemic in France, Italy, USA and China [6, 8–12]. From the end of April 2020, the number of patients admitted to ICUs in metropolitan France for coronavirus disease (COVID)-19 decreased sharply.

The second wave of the outbreak started in July 2020, accelerated significantly from the end of September 2020, and was coming to an end by late December 2020. To date, data regarding the characteristics, management, and prognosis of patients admitted to ICUs during this second wave of the outbreak in France are scarce [13], but they may differ to those from the first wave. First, during the second wave, ICUs were not as close to being overwhelmed as during the first wave. This is of importance since strain on critical care capacities is associated with mortality [14]. Second, patient management was improved, with the demonstration of the benefit of corticosteroid therapy [15, 16] and of non-invasive strategies of oxygenation [17–19].

The first aim of the present retrospective multicenter study was to describe the characteristics, management, and prognosis of COVID-19 patients admitted to ICUs during the second wave of the outbreak in France and to compare them with the first wave. The second aim was to evaluate the relationship between ICU strain and mortality.

## Patients and methods

### Study design, patients

This retrospective observational study was performed in five hospitals, four in the Paris area and one in eastern France. Based on ICU discharge diagnostic code and on laboratory database, all consecutive patients with laboratory-confirmed SARS-CoV-2 infection admitted to one of the ICUs between 21 February and 31 December 2020, were enrolled. Laboratory confirmation of SARS-Cov-2 was defined as a positive result of real-time reverse transcriptase–polymerase chain reaction (RT-PCR) assay of nasal and pharyngeal swabs. The study was approved by the ethics committee of the French Intensive Care Society (n. CE SRLF 20–89), which waived the need for informed consent from individual patients due to the retrospective nature of the study. The study complied with the Strengthening the Reporting of Observational Studies in Epidemiology (STROBE) Statement guidelines (http://www.equator-network.org). Main characteristic of the five participating centers are displayed on S1 Table.

### Data collection

Data were recorded by in-charge intensivists at each hospital from medical records and electronic reports.

Baseline information collected at ICU admission were: date of ICU admission, age, gender, body mass index (BMI), date of the first symptom, dates of hospital and ICU admission and comorbidities. Regarding comorbidities, chronic obstructive pulmonary disease was defined as previously reported [20], overweight was defined by a BMI >25 kg.m^-2^ and obesity was defined by a BMI >30 kg.m^-2^. Immunosuppression included patients with solid tumors, hematological malignancies, solid organ transplantation, long-term immunosuppressive drugs, or HIV infection [21]. Chronic kidney disease (CKD) was defined as stage 2 to 5 according to the Kidney Disease: Improving Global Outcomes (KDIGO) guidelines [22]. The following were also collected on the day of admission: body temperature, ratio of arterial oxygen tension to inspired oxygen fraction (PaO_2_/FiO_2_) at day 1 (worst value, calculated by converting O_2_ flow to estimated FiO_2_ [23]), lactate, leucocytes, platelet, fibrinogen, ferritin, and Sequential Organ Failure Assessment (SOFA) calculated within 24 hours of ICU admission [24]. During the ICU stay, oxygen strategy was recorded (standard oxygen, high flow nasal cannula [HFNC], continuous positive airway pressure [CPAP], or non-invasive ventilation [NIV]), need for endotracheal intubation, administration of corticosteroids and tocilizumab, acute kidney injury [25], need for renal replacement therapy, vasopressors, extracorporeal lung support and tracheostomy. The occurrence of ventilator-associated pneumonia was also collected.

Finally, the following outcomes were recorded: ICU mortality, hospital mortality, mortality at day 28 and day 60, ICU length of stay, and hospital length of stay.

Strain on ICU beds capacity (Strain) was measured as follows. To allow comparability across centers independently from baseline number of ICU beds, strain was scaled for every of the centers. Briefly, strain reflects number of COVID-19 patients in each ICU at a dedicated month, when compared to mean number of patients admitted as a mean each month in the same center. This result is reported in standard deviation (SD). Therefore, a strain of +1 reflects 1 SD increase in monthly COVID-19 admission, when compared to mean number of admission during study period in the same center.


$$ICU\;strain\;for\;a\;given\;month=\frac{number\;of\;COVID\;admission–mean\;number\;of\;COVID\;admission}{standard\;deviation\;of\;admission}$$

### Statistical analysis

Continuous variables were described as median (interquartile range) and compared between groups using the non-parametric Wilcoxon rank-sum test. Categorical variables were described as frequency (percentages) and compared between groups using Fisher’s exact test. Mortality was assessed using survival analysis.

Patients were grouped according to the COVID-19 pandemic wave during which they were admitted to the ICU. The first wave was defined as the period from 21 February to 30 June 2020 and the second wave was defined as the period from 1^st^ July to 31 December 2020.

Independent risk factors of day 28 mortality were assessed using mixed logistic regression with center as random effect on the intercept. To avoid overfitting, conditional stepwise variable selection was performed with 0.2 as the critical P-value for entry into the model, and 0.1 as the P-value for removal. Interactions and correlations between the explanatory variables were carefully checked. It was preplanned to assess assumption for log-linearity of continuous variables, and if not met, to transform continuous variables into quartiles.

Overall, rate of missing data was 7.4% and the rate of missing data among major outcomes or covariates was <0.1%. No imputation of missing data was performed.

Our goal was to show a decrease in mortality from 35% during the first wave as reported by many studies, to 26% during the second wave. Given the time point at which we conducted the study, we anticipated that 55% of patients would be admitted during the first wave and 45% during the second wave. With a type 1 error rate of 0.05 and a type 2 error rate of 0.10, a sample of 1084 patients was needed.

Statistical analyses were performed with R statistical software, version 4.0.5 (available online at http://www.r-project.org/), and ‘lme4’ and ‘lmerTest’ packages were used. A P value <0.05 was considered significant.

## Results

**Fig 1:**
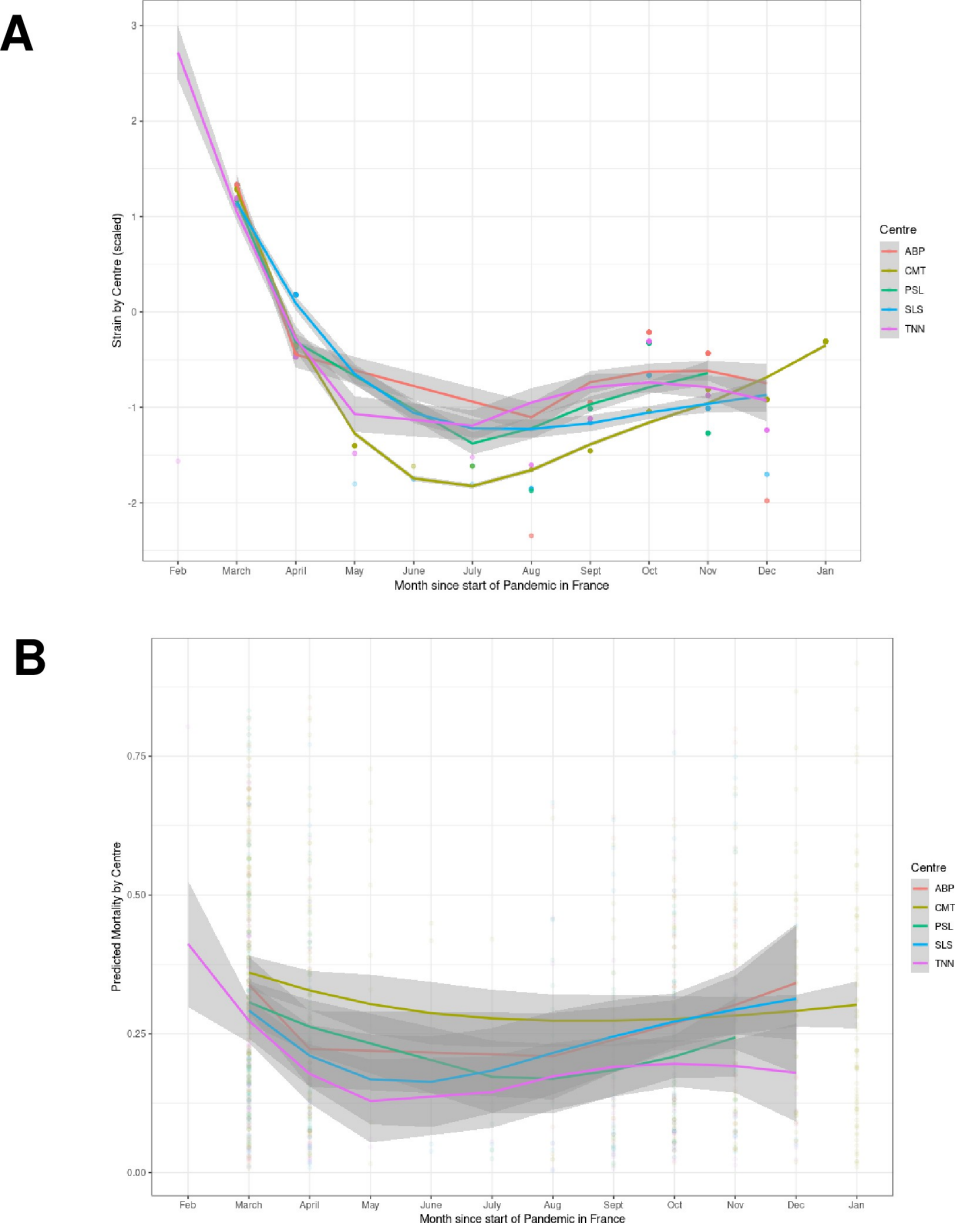
Evolution of intensive care unit (ICU) strain (Panel A) and predicted mortality (Panel B) over the entire study period. ICU strain within center was calculated as follows: (number of COVID-19 admission in each center–mean number of COVID-19 admission in each center during the whole period) / standard deviation of admission in each center. Bold line and gray area represent respectively mean by center and 95% confidence interval.

Overall, 1166 patients were admitted to the five participating ICUs, 640 (55%) during the first wave and 526 (45%) during the second wave (see S1 Fig. for the study flow chart). Fig 1A shows the evolution of strain over the entire study period. The ICU strain was lower during the second wave than the first wave (-0.81 [-1.04 –-0.31] vs. 1.18 [-0.34–1.29], P<0.001).

Tables 1 and 2 show the main characteristics of patients at ICU admission and patient management and outcome according to the wave of the pandemic during which they were admitted to the ICU. Those admitted during the second wave were older than those admitted during the first wave (median [inter-quartile range (IQR)]: 67 [58–73] vs. 63 [55–71] years, P<001), but no difference was observed in term of comorbidities. In term of severity, patients admitted during the second wave had more profound hypoxemia (PaO_2_/FIO_2_ 96 [70–147] vs. 118 [81–181] mmHg, P<0.001) but were less likely to require vasopressor administration (29% vs. 39%, P<0.001) or acute kidney injury (44% vs. 57%, P<0.001), and a smaller proportion needed renal replacement therapy (10% vs. 17%, P<0.001) during the ICU stay. Patients admitted during the second wave had a lower SOFA score (3 [2–6] vs. 4 [3–7], P<0.001). In term of oxygenation and ventilation strategy, HFNC was more frequently used during the second wave (68% vs. 39%, P<0.001), as was NIV (23% vs. 17%, P = 0.009). CPAP use was unchanged. Overall, the proportion of patients who were intubated during their ICU stay decreased during the second wave (46% vs. 69%, P<0.001) and the time between ICU admission and intubation increased (1 [0–2] days vs. 0 [0–1] days, P<0.001). The use of steroids was higher in the second wave than in the first wave (95% vs. 31%, P<0.001). The proportion of patients who developed ICU-acquired pneumonia was not different between the two periods (29% vs. 29%, P = 0.42). The need for extracorporeal lung support (3% vs. 6%, P = 0.04) and tracheostomy (4% vs. 14%, P<0.001) decreased during second wave.

**Table 1:** Main characteristics at ICU admission.

	First wave (n = 640)	Second wave (n = 526)	P Value
ICU strain, *change in SD in each center*	1.18 (-0.34–1.29)	-0.81 (-1.04 –-0.31)	<0.001
**Patient characteristics**			
Age, *years*	63 (55–71)	67 (58–73)	<0.001
Male, *n (%[95 CI])*	481 (75 [72–78])	371 (71 [66–74])	0.09
Body mass index, *kg*.*m*^*-2*^	28.0 (24.8–32.2)	28.7 (25.2–32.8)	0.22
*Comorbidities*			
Body weight including			0.29
*Overweight*, *n (%[95 CI])*	221 (35 [31–38])	178 (34 [30–38])	
*Obesity*, *n (%[95 CI])*	222 (35 [31–38])	204 (39 [35–43])	
COPD, *n (%[95 CI])*	50 (8 [6–10])	50 (10 [7–12])	0.36
Asthma, *n (%[95 CI])*	34 (5 [4–7])	28 (5 [4–8])	1.00
High blood pressure, *n (%[95 CI])*	328 (51 [47–55])	267 (51 [46–55])	0.91
Diabetes, *n (%[95 CI])*	189 (30 [26–33])	160 (30 [27–35])	0.79
Immunosuppression, *n (%[95 CI])*	88 (14 [11–16])	77 (15 [12–18])	0.73
Chronic heart failure, *n (%[95 CI])*	43 (7 [5–9])	39 (7 [5–10])	0.73
Chronic kidney disease, *n (%[95 CI])*	77 (12 [10–15])	54 (10 [8–13])	0.39
**On ICU admission**			
Time since disease onset, *days*	8 (6–11)	9 (6–11)	0.15
Time since hospital admission, *days*	1 (0–1)	1 (0–3)	0.003
SOFA	4 (3–7)	3 (2–6)	<0.001
Body temperature, *°C*	38.2 (37.4–39.0)	37.7 (37.1–38.5)	<0.001
PaO_2_/FiO_2_ on the first day following ICU admission (worst value), *mmHg*	118 (81–181)	96 (70–147)	<0.001
Lactate, *mMol*.*L*^*-1*^	1.50 (1.20–2.00)	1.60 (1.20–2.12)	0.08
Leucocytes, *G*.*L*^*-1*^	8.5 (6.0–11.5)	8.7 (6.3–11.7)	0.24
Platelet, *G*.*L*^*-1*^	209 (158–270)	220 (165–290)	0.02
Fibrinogen, *g*.*L*^*-1*^	6.9 (5.9–7.8)	7.00 (5.8–7.9)	0.53
Ferritin, *μg*.*L*^*-1*^	1414 (797–2454)	1143 (582–1981)	0.01
Serum Creatinine, *μmol*.*L*^*-1*^	82 (65–116)	78 (61–113)	0.07

ICU strain within center was calculated as follows: (number of COVID-19 admission in each center–mean number of COVID-19 admission in each center during the whole period) / standard deviation of admission in each center (see Patients and Methods section).

ICU, intensive care unit; SD, standard deviation; COPD, chronic obstructive pulmonary disease; PaO_2_/FiO_2_, ratio of arterial oxygen tension to inspired oxygen fraction; SOFA, Sequential Organ Failure Assessment score.

Continuous variables are expressed as median (interquartile range) and categorical variables as absolute value (% [95% confidence interval]).

**Table 2:** Management and outcomes.

	First wave (n = 640)	Second wave (n = 526)	P Value
**Oxygenation/ventilation strategy**			
HFNC, *n (%[95 CI])*	250 (39 [35–43])	362 (68 [65–63])	<0.001
CPAP, *n (%[95 CI])*	13 (2 [1–3])	10 (2 [1–3])	1.00
NIV, *n (%[95 CI])*	106 (17 [14–20])	120 (23 [19–27])	0.009
**Immunomodulatory therapy**			
Steroids, *n (%[95 CI])*	195 (31 [27–34])	498 (95 [92–96])	<0.001
Tocilizumab, *n (%[95 CI])*	23 (4 [2–5])	3 (1 [0–1])	0.001
**Organ failure and support during ICU stay**			
Acute kidney injury stage			<0.001
0, *n (%[95 CI])*	272 (43 [39–46])	292 (56 [51–60])	
1, *n (%[95 CI])*	266 (42 [38–45])	158 (30 [26–34])	
2, *n (%[95 CI])*	37 (6 [4–8])	33 (6 [4–9])	
3, *n (%[95 CI])*	65 (10 [8–13])	41 (8 [6–10])	
Vasopressors within first 24 hours, *n (%[95 CI])*	252 (39 [36–43])	151 (29 [25–33])	<0.001
Renal replacement therapy, *n (%[95 CI])*	111 (17 [14–21])	52 (10 [7–13])	<0.001
Extracorporeal lung support, *n (%[95 CI])*	40 (6 [5–8])	18 (3 [2–5])	0.04
Tracheotomy, *n (%[95 CI])*	91 (15 [12–17])	23 (4 [3–6])	<0.001
**ICU-acquired pneumonia, *n*** *(%[95 CI])*	188 (29 [26–33])	155 (29 [25–33])	0.97
**Outcome variables**			
Invasive mechanical ventilation at day 28, *(%[95 CI])*	444 (69 [66–73])	243 (46 [42–50])	<0.001
Time from ICU admission to initiation of mechanical ventilation, *days*	0 (0–1)	1 (0–2)	<0.001
Duration of mechanical ventilation, *days*	13 (7–20)	12 (2–21)	0.50
ICU mortality, *n (%[95 CI])*	200 (31 [28–35])	144 (28 [42–51])	0.18
Hospital mortality, *n (%[95 CI])*	209 (34 [29–36])	159 (31 [26–34])	0.27
Mortality at day 28, *n (%[95 CI])*	192 (30 [26–34])	135 (26 [22–30])	0.12
Mortality at day 60, *n (%[95 CI])*	214 (33 [30–37])	161 (31 [27–35])	0.35
ICU length of stay, *days*	9 (4–18)	9 [5–18]	0.78
Hospital length of stay, *days*	15 (8–27)	17 (11–27)	0.009

HFNC, high flow nasal cannula; CPAP, continuous positive airway pressure; NIV, non-invasive ventilation; ICU, intensive care unit.

Continuous variables are expressed as median (interquartile range) and categorical variables as absolute value (% [95% confidence interval]).

**Fig 2:**
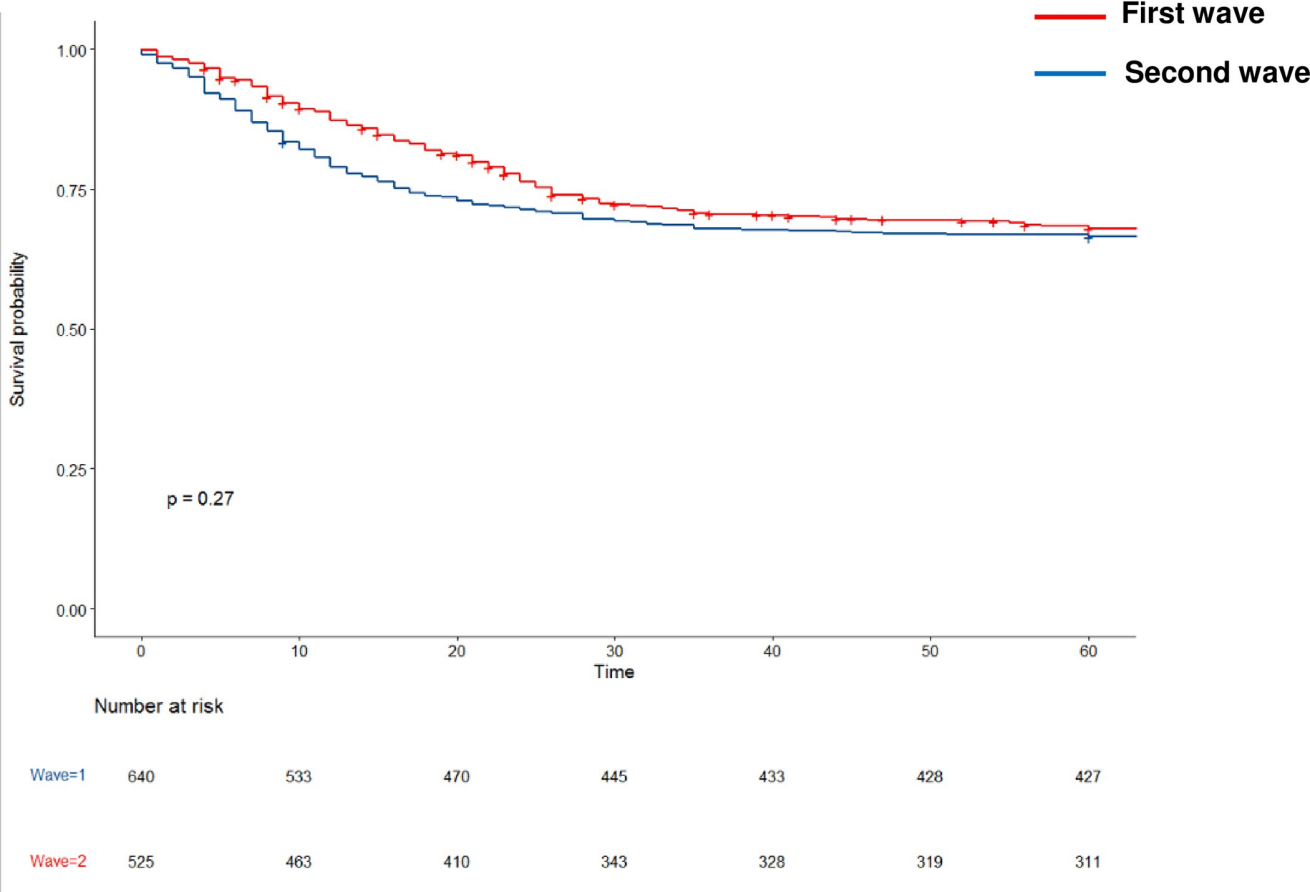
Cumulative incidence of death during the first and the second wave.

Overall, day 28 and hospital mortality was 28% (n = 327) and 32% (n = 368), respectively (Table 2). Neither day 28 mortality (30% vs. 26%) nor hospital mortality (33% vs. 31%) differed between first and second wave (P = 0.12 and P = 0.27, respectively). Cumulative incidence of death was not different between the first and the second wave (log rank test, p = 0.22, Fig 2).

Table 3 shows the factors associated with 28-day mortality by univariate analysis. After adjustment for confounders, including random effect on center on the intercept, five variables were independently associated with 28-day mortality (area under the curve of the model 0.76, 95% CI 0.73–0.79). Overweight and obesity (respectively odds ratio [OR] 0.69; 95% confidence interval [CI] 0.48–0.98, and 0.59; 95% CI 0.41–0.85) were associated with lower mortality when compared to BMI <25 kg.m^-2^. Four variables were associated with higher mortality: older age (OR 1.07 per year; 95% CI 1.05–1.08), underlying chronic kidney disease (OR 1.74; 95% CI 1.13–2.69), severity at ICU admission as assessed by SOFA score (OR 1.22 per point; 95% CI 1.17–1.28) and strain per center (OR 1.20 per change in SD; 95% CI 1.04–1.39). Underlying high blood pressure remained in the model without reaching statistical significance (OR 1.29; 95% CI 0.95–1.74) (see also S2 Table for the detailed final model of the multivariate analysis). Regarding hospital mortality, by multivariate analysis, association between strain per center and mortality was OR 1.12 per change in SD (95% CI 0.98–1.28).

**Table 3:** Factors associated with mortality 28 days after admission to the intensive care unit: Univariate and multivariate analysis.

	Day 28 Survivors (n = 839)	Day 28 Non-survivors (n = 327)	P Value	Multivariate analysis Odds ratio (95% confidence interval) (n = 1163)
Admissions during the second wave, *n (%)*	390 (47)	135 (41)	0.120	
ICU strain, *Change in SD in each center*	-0.33 (-0.87–1.18)	-0.31 (-0.81–1.19)	0.017	1.20 per change in SD (1.04–1.39)
**Patients characteristics**				
Age, *years*	62 (54–70)	70 (63–76)	<0.001	1.07 (1.05–1.08)
Male gender, *n (%)*	597 (71)	255 (78.0)	0.024	
Body mass index, *kg*.*m*^*-2*^	28.6 (25.4–32.9)	27.6 (24.5–31.5)	0.010	
*Comorbidities*				
Overweight, *n (%)*	289 (35)	110 (34)	0.784	0.69 (0.48–0.98)
Obesity, *n (%)*	323 (39)	102 (31)	0.019	0.59 (0.41–0.85)
COPD, *n (%)*	48 (6)	52 (16)	<0.001	
Asthma, *n (%)*	55 (7)	7 (2)	0.004	
High blood pressure, *n (%)*	393 (47)	201 (62)	<0.001	1.29 (0.95–1.74)
Diabetes, *n (%)*	227 (27)	121 (37)	0.001	
Immunosuppression, *n (%)*	115 (14)	50 (15)	0.551	
Chronic heart failure, *n (%)*	43 (5)	39 (12)	<0.001	
Chronic kidney disease, *n (%)*	74 (9)	57 (17)	<0.001	1.74 (1.13–2.69)
**On ICU admission**				
Time since disease onset, *days*	9 (6–11)	8 (6–10)	0.001	
Time since hospital admission, *days*	1 (0–2)	1 (0–2)	0.452	
Body temperature, *°C*	38.0 (37.2–38.7)	37.9 (37.2–38.8)	0.819	
PaO_2_/FiO_2_ at day 1 (worst value), *mmHg*	115 (78–174)	99 (72–145)	0.001	
Lactate, *mMol*.*L*^*-1*^	1.5 (1.2–2.0)	1.6 (1.2–2.3)	0.001	
Leucocytes, *G*.*L*^*-1*^	8.4 (6.1–11.3)	8.8 (5.6–12.4)	0.239	
Platelet, *G*.*L*^*-1*^	223 (168–290)	184 (140–245)	<0.001	
Fibrinogen, *g*.*L*^*-1*^	7.0 (5.9–7.9)	6.8 (5.8–7.8)	0.329	
Ferritin, *μg*.*L*^*-1*^	1230 (687–2132)	1663 (799–3016)	0.035	
SOFA	3 (2–6)	6 (4–8)	<0.001	1.22 (1.17–1.28)
**Oxygenation/ventilation strategy**				
HFNC, *n (%)*	451 (54)	160 (49)	0.15	
CPAP, *n (%)*	17 (2)	6 (2)	1.00	
NIV, *n (%)*	156 (19)	70 (21)	0.32	
**Immunomodulatory therapy**				
Steroids, *n (%)*	506 (60)	186 (57)	0.304	
Tocilizumab, *n (%)*	22 (3)	4 (1)	0.217	
**Organ failure and support during ICU stay**				
Invasive mechanical ventilation at day 28, *n (%)*	437 (52)	250 (77)	<0.001	
Acute kidney injury stage			<0.001	
0, *n (%)*	479 (57)	84 (26)		
1, *n (%)*	265 (32)	159 (49)		
2, *n (%)*	47 (6)	23 (7)		
3, *n (%)*	46 (6)	60 (18)		
Vasopressors, *n (%)*	239 (29)	164 (50)	<0.001	
Renal replacement therapy, *n (%)*	78 (9)	85 (26)	<0.001	
**ICU-acquired pneumonia, *n (%)***	241 (39)	102 (37)	0.178	

ICU strain within center was calculated as follows: (number of COVID-19 admission in each center–mean number of COVID-19 admission in each center during the whole period) / standard deviation of admission in each center (see Patients and Methods section).

ICU, intensive care unit; SD, standard deviation; COPD, chronic obstructive pulmonary disease; PaO_2_/FiO_2_, ratio of arterial oxygen tension to inspired oxygen fraction; SOFA, Sequential Organ Failure Assessment score; HFNC, high flow nasal cannula; CPAP, continuous positive airway pressure; NIV, non-invasive ventilation.

Continuous variables are expressed as median (interquartile range) and categorical variables as absolute value (%).

**Fig 3:**
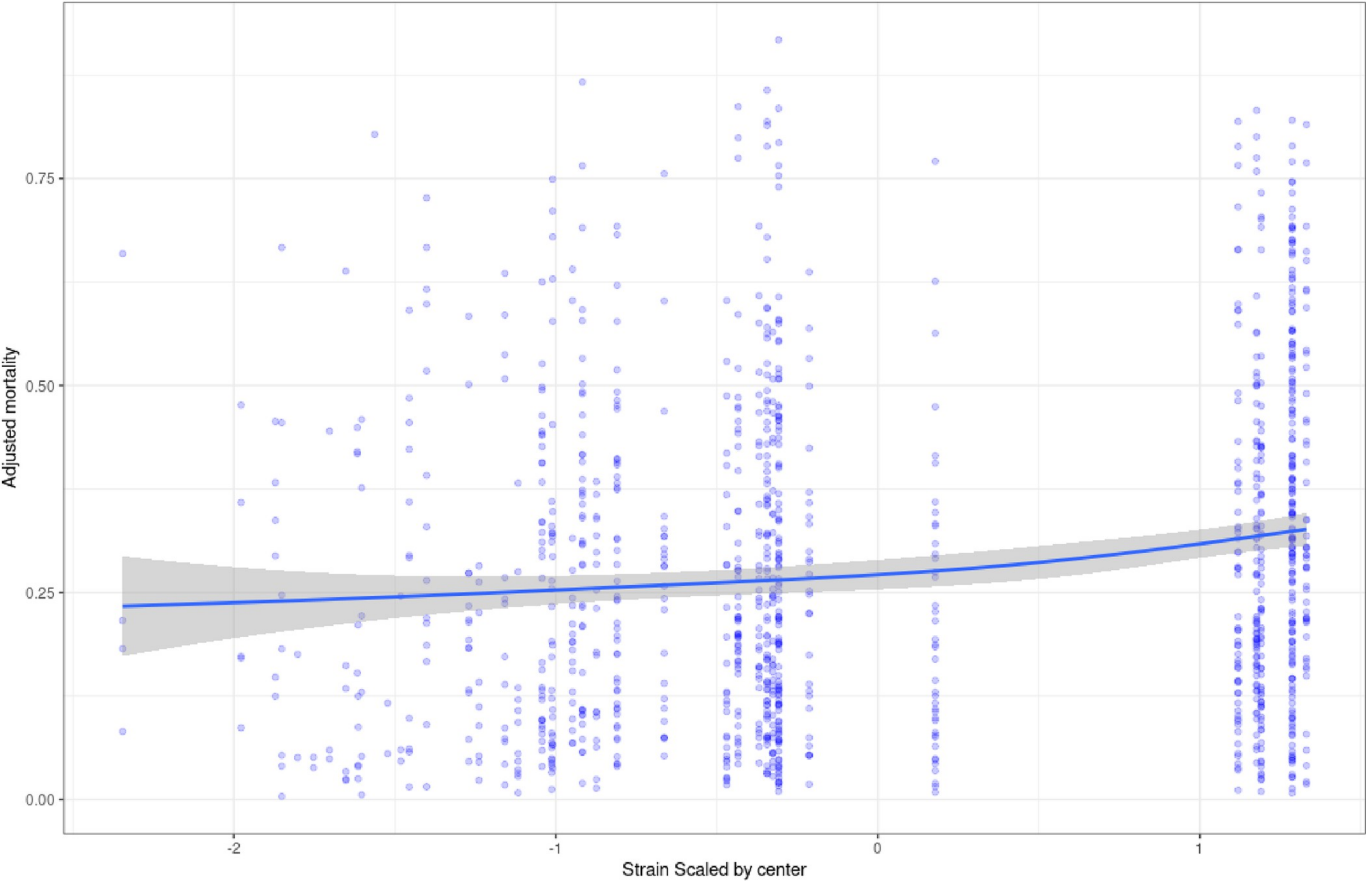
Adjusted predicted mortality as a function of intensive care unit (ICU) strain. ICU strain within center was calculated as follows: (number of COVID-19 admission in each center–mean number of COVID-19 admission in each center during the whole period) / standard deviation of admission in each center. Bold line and gray area represent respectively fit and 95%CI.

Fig 1B shows changes in predicted mortality over time. Overall change in predicted mortality as a function of strain is reported in Fig 3.

Mortality was adjusted on the variables that remained in the final multivariate model: age, high blood pressure, overweight, obesity, chronic kidney disease, Sequential Organ Failure Assessment.

## Discussion

The two main findings of this study are as follows. First, patients admitted during the second wave differed to those admitted during the first wave. Particularly, they were older and less severely ill in term of organ failure. In addition, they received steroids and HFNC more frequently and were less frequently intubated. Second, mortality was not different between the first and the second wave, but was independently associated with ICU strain. These findings may guide bed management during future COVID-19 waves.

In term of mortality, we did not observe a decrease between the two waves. This might be explained by the fact that improvements were made regarding mortality within the first wave. For instance, in a large cohort in France, Belgium and Switzerland, mortality decreased from 42% in late March 2020 to 25% in early May 2020 [8]. In terms of patients characteristics and management, factors associated with mortality were similar to those previously described [8–10], with the exception of obesity that we found to be associated with lower mortality, which has not been commonly observed in the COVID [26]. It is of note that we found an independent relation between ICU strain and mortality. The hypothesis that an increase in patient needs strains hospital resources, which in turn worsens the quality of care and outcome is not new. A meta-analysis showed that in 9 of 12 cohort studies in ICU settings between 1999 and 2015, mortality increased during periods of strain on capacity [27]. In the context of the COVID-19 pandemic, similar observations have been made in Veterans Affairs hospitals. A cohort study of 8516 patients with COVID-19 admitted to 88 hospitals found that although facilities augmented ICU capacity during the pandemic, strains on critical care capacity were associated with increased COVID-19 ICU mortality [14]. In France, a retrospective national surveillance analysis showed that the probability of death was significantly correlated with COVID-19 ICU occupancy [28]. As opposed to this study, the sample size of our study was smaller. Nevertheless, our study provided more detailed information on patients. As opposed to risk factors for mortality such as age or comorbidities, strain on the ICU is more manageable, and a policy aiming to increase ICU bed availability could in turn reduce the strain on the ICU. Such a policy should consider the wide variation of geographical access to ICU beds across European countries, with low ICU accessibility being associated with a higher proportion of COVID-19 deaths [29].

Patients admitted during the second wave were older than those admitted during the first wave. Although this might be due to the virus itself, one cannot exclude that the lower ICU strain during this second wave led physicians to be less restrictive on age when considering new ICU admission. Interestingly, in term of comorbidity, there was no difference between patients admitted during the two first waves, suggesting that the profile of patients admitted for a severe form of COVID-19 did not change throughout the study period. Hypoxemia on ICU admission was more pronounced in patients admitted during the second wave. The increase in time from hospital admission to ICU admission may suggest that patients were transferred to the ICU later and were therefore at a more hypoxemic stage of the disease during the second wave of the outbreak.

In terms of management, we observed noticeable changes between the first and second waves. First, corticosteroids were given to the vast majority (95%) of patients during the second wave, while only 31% of patients received steroids during the first wave. This followed the results of the Recovery study and the subsequent meta-analysis [15, 16]. While the proportion of patients requiring invasive mechanical ventilation decrease, the proportion of patients developing an ICU-acquired pneumonia did not change, which is surprising. This might be explained by the fact that, during the second wave, more patients received steroids, which may have increased the risk of ICU-acquired pneumonia, even if less patients were intubated. Second, the proportion of patients receiving HFNC increased dramatically between the two waves. In June and July 2020, reports suggesting that HFNC could be associated with a reduced risk of intubation were published, although the benefit of HFNC on mortality remains disputed [8, 17, 30]. This increased use of HFNC had already started within the first wave of the epidemic. In a large cohort of 4244 patients, HFNC was used in about 15% of ICU patients before 15 March 2020 and in more than 30% after 16 April 2020 [8]. Whether related or not, a decrease in intubation rate paralleled the increased use of HFNC.

Strengths of this study include its multicenter design and its conduct in two areas of France that were strongly affected by the COVID-19 pandemic. The study has several limitations. First, the design was retrospective, so the causality between ICU strain and mortality cannot be inferred. However, only few data were missing (7.4%). In addition, to date, this is one of the largest studies to compare patients admitted during the two first waves. Other studies were either of smaller size, with a lower level of data granularity, or conducted in middle income rather than in high income countries [13, 30–33]. Second, we only focused on mortality, although strain could also increase morbidity. Third, we did not precisely measure staffing nor the way facilities expanded with increased needs for ICU beds. Fourth, data on treatment limitation decisions were lacking, which is one of the multiple potential confounders of our study.

In conclusion, the characteristics and the management of patients varied between the first and the second wave of the COVID-19 pandemics, but the mortality did not change. Rather than the wave of the pandemic, ICU strain was independently associated with 28-day mortality: the higher the strain, the higher the mortality. This association was not found for hospital mortality. ICU strain is definitely a manageable factor. Subsequently, public health officials and hospital administrators should consider interventions that reduce COVID-19-related ICU strain. These interventions may aim to decrease ICU bed demand or to increase ICU bed availability and staffing.

## Supporting information

S1 Fig Flow chart of the study. (TIF)

S1 Table Main characteristics of the five participating centers. (DOCX)

S2 Table Factors associated with mortality 28 days after admission to the intensive care unit: Multivariate analysis. (DOCX)

S1 Data (CSV)
