# Supplementary figures and images for: ICU strain and outcome in COVID-19 patients—A multicenter retrospective observational study
Source: PLoS One. 2022 Jul 19;17(7):e0271358. doi: 10.1371/journal.pone.0271358 (PMC9295940; doi:10.1371/journal.pone.0271358)

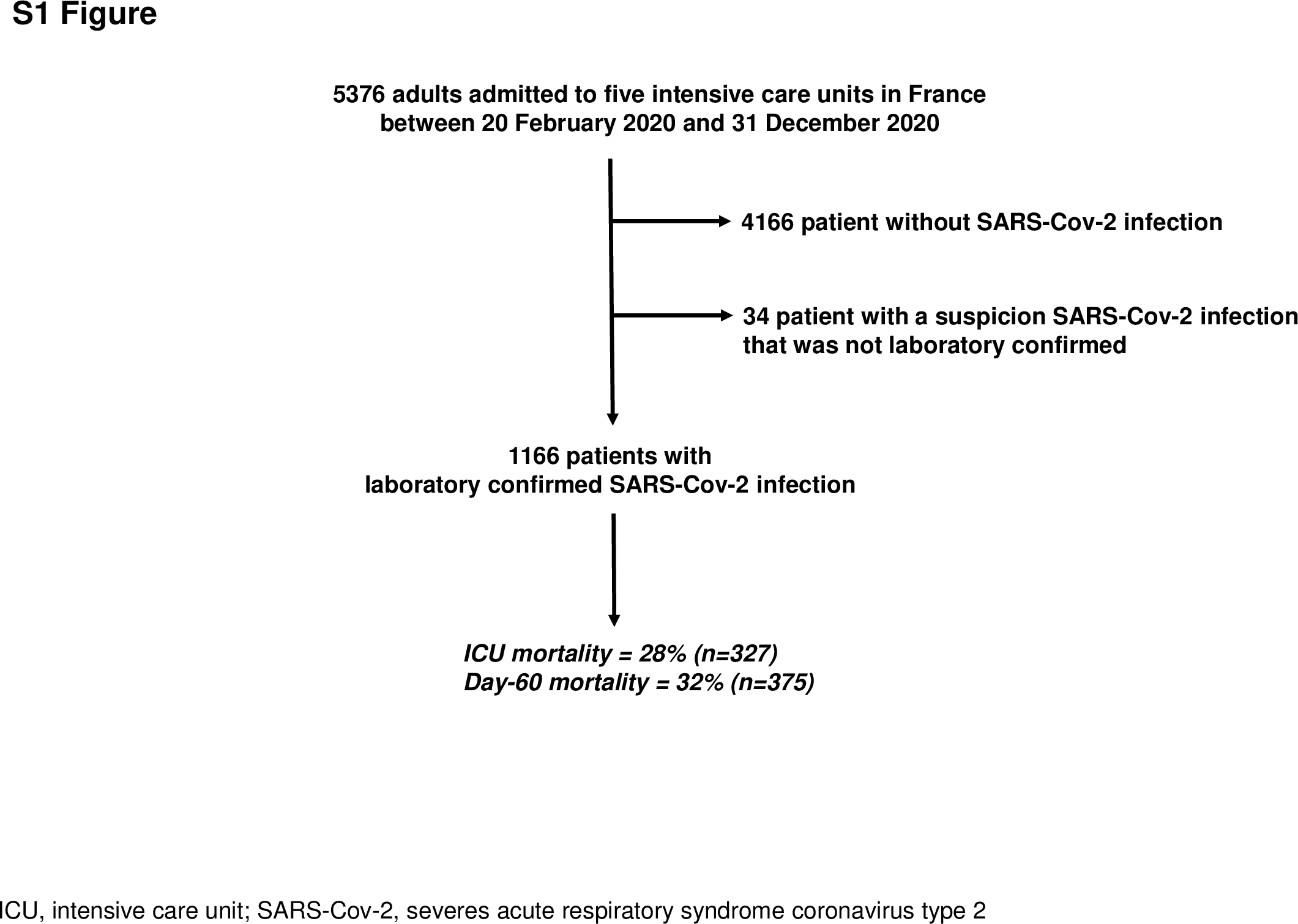

Supplement: S1 Fig — (TIF) [file pone.0271358.s001.tif]
