# Supplementary material for: ICU strain and outcome in COVID-19 patients—A multicenter retrospective observational study
Source: PLoS One. 2022 Jul 19;17(7):e0271358. doi: 10.1371/journal.pone.0271358 (PMC9295940; doi:10.1371/journal.pone.0271358)
Supplement: S1 Table — (DOCX) [file pone.0271358.s002.docx]

**S1 Table. Main characteristics of the five participating centers**

|  | **Number of beds in the hospital** | **Number of ICU beds in the hospital** | **Number of beds in the participating**  **ICU** | **Maximum number of beds in the participating ICU during the COVID-19 surges** | **Total number of patients admitted in the ICU between 20 March and 31 Decembre 2020** |
| --- | --- | --- | --- | --- | --- |
| **Centre 1** | 404 | 12 | 12 | 34 | 1284 |
| **Centre 2** | 1621 | 105 | 15 | 32 | 1131 |
| **Centre 3** | 650 | 24 | 12 | 30 | 863 |
| **Centre 4** | 525 | 15 | 15 | 42 | 799 |
| **Centre 5** | 810 | 70 | 26 | 117 | 1299 |

ICU, intensive care unit.
