# Supplementary material for: ICU strain and outcome in COVID-19 patients—A multicenter retrospective observational study
Source: PLoS One. 2022 Jul 19;17(7):e0271358. doi: 10.1371/journal.pone.0271358 (PMC9295940; doi:10.1371/journal.pone.0271358)
Supplement: S2 Table — (DOCX) [file pone.0271358.s003.docx]

**S2 Table. Factors associated with mortality 28 days after admission to the intensive care unit: multivariate analysis**

|  | **Hazard ratio**  **(95% confidence interval)** | **p** |
| --- | --- | --- |
| ICU strain, *per change in SD* | 1.20 (1.04 – 1.39) | 0.012 |
| Age, *per year* | 1.07 (1.05 – 1.08) | <0.001 |
| High blood pressure | 1.29 (0.95 – 1.74 | 0.099 |
| Overweight | 0.69 (0.48 – 0.98) | 0.039 |
| Obesity | 0.59 (0.41 – 0.85) | 0.005 |
| Chronic kidney disease | 1.74 (1.13 – 2.69) | 0.012 |
| SOFA, *per point* | 1.22 (1.17 – 1.28) | <0.001 |

ICU, intensive care unit; SD, standard deviation; SOFA, Sequential Organ Failure Assessment score.

ICU strain within center was calculated as follows: (number of COVID-19 admission in each center – mean number of COVID-19 admission in each center during the whole period) / standard deviation of admission in each center (see Patients and Methods section).
